# Supplementary material for: Magnetosheath jets at Jupiter and across the solar system
Source: Nat Commun. 2024 Jan 9;15:4. doi: 10.1038/s41467-023-43942-4 (PMC10776788; doi:10.1038/s41467-023-43942-4)
Supplement: Supplementary file 1 — Supplementary Infomation [file 41467_2023_43942_MOESM1_ESM.pdf]

Supplementary Information to "Magnetosheath Jets at Jupiter and Across the Solar System"

Yufei Zhou,[1] Savvas Raptis,[2] Shan Wang,[3] Chao Shen*,[1] Nian Ren,[1] and Lan Ma[1]

[1] *School of Science, Harbin Institute of Technology (Shenzhen), Shenzhen, China*
[2] *Applied Physics Laboratory, Johns Hopkins University, Laurel, MD, USA*
[3] *Institute of Space Physics and Applied Technology, Peking University, Beijing, China*

(Dated: November 19, 2023)

KRONIAN MAGNETOSHEATH JET

Here we present a preliminary Kronian magnetosheath jet report.

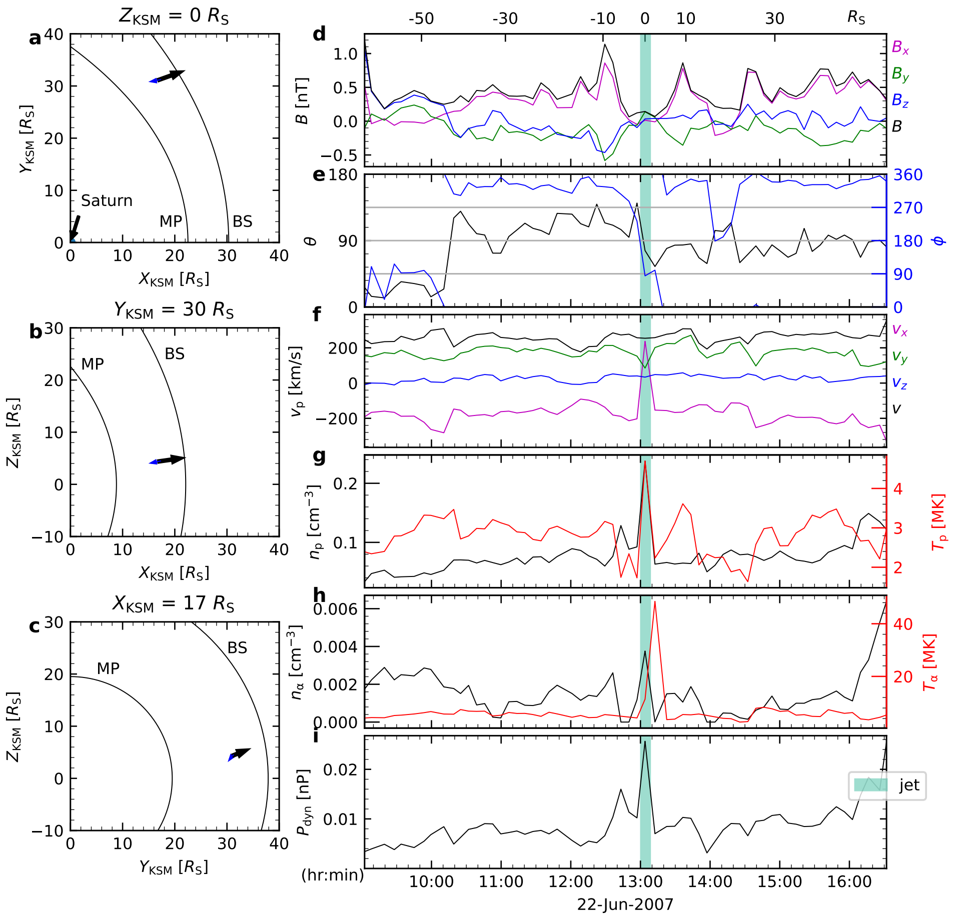

FIG. 1. Jet observation in Kronian magnetosheath. Same format as Fig. 1 in the main text. The KSM (Kronocentric Solar Magnetospheric) coordinate system is utilized. The positions of the bow shock and magnetopause are modeled using the Went11 and Kanani10 models [1, 2] with an upstream dynamic pressure of 0.02nPa. Left : (a-c) spacecraft trajectory plots in the MSO (Mars Solar Orbital) coordinates. Bow shock is modeled using [3]. Right: Color shading marks jet. The displayed quantities are magnetic field in MSO (d) Cartesian coordinates and (e) spherical coordinates; (f) proton bulk velocity; number density and temperature of (g) protons and (h) alpha particles; (i) ion dynamic pressure. The scales at the top of panel (d) indicate the distance traveled by the spacecraft within the magnetosheath flow. Source data are provided as a Source Data file.

At Saturn, the orbit of Cassini spacecraft passed through the subsolar magnetosheath between 2007 and 2008. However, continuous observations were limited due to the low temporal resolution of ion moments and frequent instances where the primary plasma flow component was outside the detector's field of view. Fig. 1 (d-i) show data from Cassini captured from 10:00 to 16:00 UTC on 2007-06-22 when the spacecraft was in the Kronian magnetosheath. Fig. 1

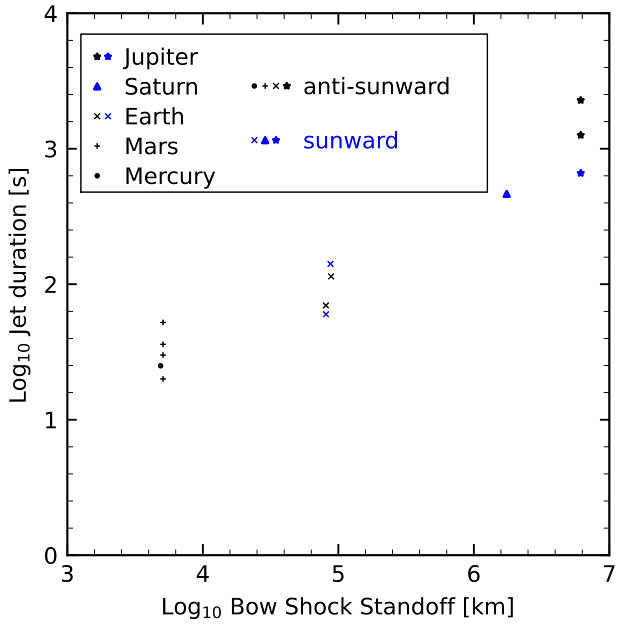

FIG. 2. Jet observation duration plotted against bow shock standoff distance for Mercury, Mars, Earth, Saturn, and Jupiter. Blue (black) markers represent sunward (anti-sunward) jets. Source data are provided as a Source Data file.

(a-c) depict the trajectory of the spacecraft in the Kronocentric-Solar-Magnetospheric (KSM) coordinate system[1, 2]. A sunward jet was encountered around 13:00 (Fig. 1 (a) and Fig. 1 (i)). Its direction is marked with a black arrow in Fig. 1 (a-c). Similar to the sunward jet observed in the Jovian magnetosheath, the Kronian jet was also associated with a weakening and rotation in magnetic field (Fig. 1 (e)) and featured by an increase in temperature of both protons and alpha particles. The enhancement in the density of the hotter component, which was alpha particles in the Kronian case, was also less intense than in the density of the cooler component. However, the speed of this jet was not enhanced as compared to the ambient flow. Its dynamic pressure resulted solely from a pulse in density. In addition, this jet was surrounded by a constant flow whose v_x did not show the "breathing" effects of magnetopause and bow shock.

Due to the low resolution of plasma measurement, the jet consisted of only one data point, which made the event less convincing. The data point might contain multiple jets. However, the magnetic field did suggest the existence of a structure of this temporal-spatial scale. In view of these considerations, we present it as a preliminary result here.

COMPARISON INCLUDING KRONIAN AND POSSIBLE MERCURIAN MAGNETOSHEATH JETS

In the Mercurian magnetosheath, previously isolated magnetic structures were also identified as possible jets, although no plasma density, bulk velocity and temperature can be determined reliably in these cases[4]. The sizes of these structures are difficult to estimate. To include a clean structure at Mercury into the comparison, we use the duration of observation directly. Note that the flow speeds across planetary magentosheath are similar. Therefore, duration can serve as a crude indicator of size. The scaling trend with shock size is illustrated in Fig. 2

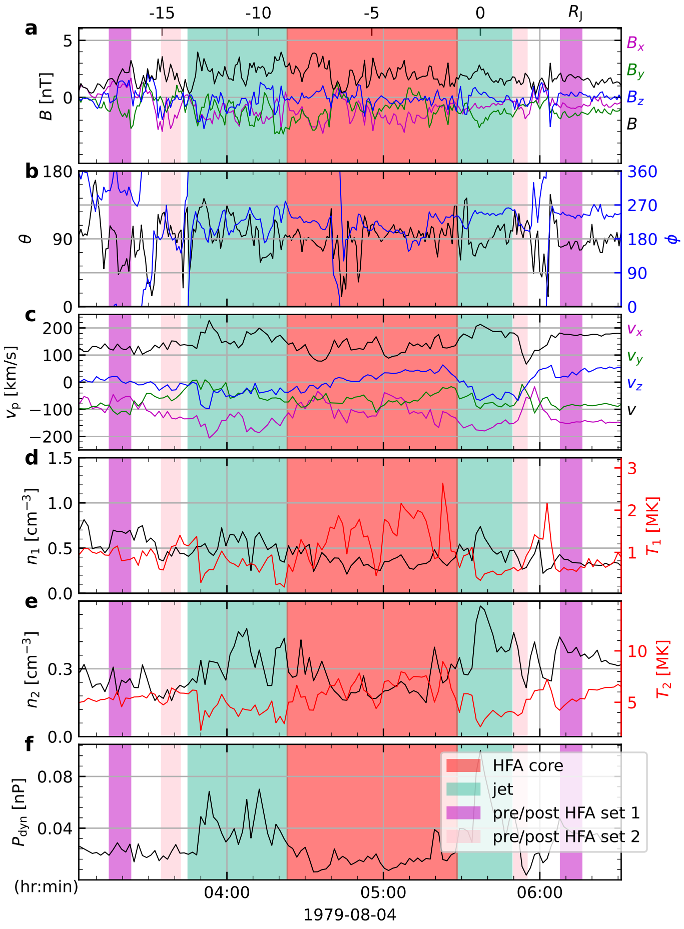

FIG. 3. Same format as Fig. 2 in the main text. The displayed quantities are magnetic field in JSO (Jupiter-Sun-Orbit) (a) Cartesian coordinates and (b) spherical coordinates; (c) ion bulk velocity; number density and temperature of the (d) cold ion component and the (e) hot ion component; (f) ion dynamic pressure. The scales at the top of panel (a) indicate the distance traveled by the spacecraft within the magnetosheath flow. Magenta and pink color shadings delineate two possible sets of pre/post-HFA data used for calculating the tangential discontinuity normal. Source data are provided as a Source Data file.

SUPPLEMENTARY METHODS

Determination of HFA Normal by Cross Product of Magnetic Field Vectors

Most HFAs are produced by shock interaction with tangential discontinuities, whose normal direction can be obtained by taking the cross product of magnetic field vectors before and after the structure [5]:

$$\mathbf{n} = \pm \frac{\mathbf{B}_{\rm pre} \times \mathbf{B}_{\rm post}}{|\mathbf{B}_{\rm pre} \times \mathbf{B}_{\rm post}|} \tag{1}$$

Magenta and pink color shading in Fig. 3 show two possible sets of pre-discontinuity and post-discontinuity regions, which result in the magnetic field conditions and discontinuity parameters shown in Tab. I. The first set (magenta) was so chosen outside of the entire event and not to reach the rotations of magnetic field around 03:30 (hr:min) and 06:00, which are visible in the spherical coordinates of magnetic field vector shown in Fig. 3 (b). This set results in a large magnetic shear angle of 73.1°. The second set (pink) was chosen to be near the event, which gave a relatively small shear angle of 33.0°. The normal directions of the discontinuity calculated from both sets are close to that obtained from MVAB method (16° ∼ 17°).

TABLE I. magnetic field conditions and discontinuity parameters.

Parameter	Value
$\mathbf{n}_{\mathrm{MVAB}}$	[-0.171, 0.040, 0.984]
Set 1 (magenta color shading in Fig. 3)	
$\mathbf{B}_{\mathrm{pre}}$	[1.09, -0.994, 0.670] nT
$\mathbf{B}_{\mathrm{post}}$	[-0.819, 1.56, 0.270] nT
$\theta_{\mathrm{BpreBpost}}$	73.1°
$\mathbf{n}_{\mathrm{BcrossB}}$	[-0.281, 0.305, 0.910]
Normal angle difference from $\mathbf{n}_{\mathrm{MVAB}}$	17.0°
Set 2 (pink color shading in Fig. 3)	
$\mathbf{B}_{\mathrm{pre}}$	[-1.78, -0.232, -0.562] nT
$\mathbf{B}_{\mathrm{post}}$	[-0.960, -0.870, -0.481] nT
$\theta_{\mathrm{BpreBpost}}$	33.0°
$\mathbf{n}_{\mathrm{BcrossB}}$	[-0.267, -0.224, 0.937]
Normal angle difference from $\mathbf{n}_{\mathrm{MVAB}}$	16.4°

Data at Saturn

Cassini data was used for Kronian jets. The magnetic field from Cassini was measured using fluxgate magnetometer (FGM)[6]. The plasma data was measured using the Cassini Plasma Spectrometer (CAPS)[7]. Cassini was also a three-axis stabilized spacecraft. The plasma detectors are mounted on an actuator which can rotate 180° thus providing a 2π field of view in one scan of roughly three minutes. The distribution function thus measured was also partial. The moments are obtained by assuming that the distribution function has mirror symmetry with respect to the bulk velocity[8]. The highest resolution of moments is $\sim$ 6 minutes. Since the proton was the dominant component, the dynamic pressure was calculated as $P_{\mathrm{dyn}} = (n_{\mathrm{p}}) m_{\mathrm{p}} v_{\mathrm{p}}^2$.

[1] D. R. Went, G. B. Hospodarsky, A. Masters, K. C. Hansen, and M. K. Dougherty, Journal of Geophysical Research: Space Physics **116**, A07202 (2011).

[2] S. J. Kanani, C. S. Arridge, G. H. Jones, A. N. Fazakerley, H. J. McAndrews, N. Sergis, S. M. Krimigis, M. K. Dougherty, A. J. Coates, D. T. Young, K. C. Hansen, and N. Krupp, Journal of Geophysical Research: Space Physics **115**, A06207 (2010).

[3] J. R. Gruesbeck, J. R. Espley, J. E. P. Connerney, G. A. DiBraccio, Y. I. Soobiah, D. Brain, C. Mazelle, J. Dann, J. Halekas, and D. L. Mitchell, Journal of Geophysical Research: Space Physics **123**, 4542 (2018).

[4] T. Karlsson, E. Liljeblad, A. Kullen, J. M. Raines, J. A. Slavin, and T. Sundberg, Planet. Space Sci. **129**, 61 (2016).

[5] S. J. Schwartz, in *Analysis Methods for Multi-Spacecraft Data*, edited by G. Paschmann and Patrick W. Daly (ESA Publications Division, Noordwijk, The Netherlands, 1998) p. 249.

[6] M. K. Dougherty, S. Kellock, D. J. Southwood, A. Balogh, E. J. Smith, B. T. Tsurutani, B. Gerlach, K.-H. Glassmeier, F. Gleim, C. T. Russell, G. Erdos, F. M. Neubauer, and S. W. H. Cowley, Space Science Reviews **114**, 331 (2004).

[7] R. J. Wilson, F. Crary, L. K. Gilbert, D. B. Reisenfeld, J. T. Steinberg, and R. Livi, (2012).

[8] M. F. Thomsen, D. B. Reisenfeld, D. M. Delapp, R. L. Tokar, D. T. Young, F. J. Crary, E. C. Sittler, M. A. McGraw, and J. D. Williams, Journal of Geophysical Research: Space Physics **115**, A10220 (2010).
