# Supplementary material for: Magnetosheath jets at Jupiter and across the solar system
Source: Nat Commun. 2024 Jan 9;15:4. doi: 10.1038/s41467-023-43942-4 (PMC10776788; doi:10.1038/s41467-023-43942-4)
Supplement: Supplementary file 3 — Description of Additional Supplementary Files [file 41467_2023_43942_MOESM3_ESM.pdf]

Description of Additional Supplementary Files

File Name: Supplementary Software 1
Description: Program "mvab", used in the study to calculate the minimum variance direction of the HFA core.
